# HIV, Stigma, and Rates of Infection: More Complicated than Reidpath and Chan Suggest

**DOI:** 10.1371/journal.pmed.0040051

**Published:** 2007-01-30

**Authors:** Sam Singer

In their essay in October's *PLoS Medicine*, Daniel Reidpath and Kit Yee Chan challenge the widely cited link between HIV-related stigma and the spread of the epidemic [1]. This is an important question, given the heavy emphasis on stigma in policies of the World Health Organization, the Joint United Programme on HIV/AIDS, and other public health institutions, but in making their argument Reidpath and Chan misrepresent the connections that other authors have made between stigma and viral transmission, ignore evidence that does suggest an association, and propose a model of their own for which they offer no evidence.

HIV infection establishes itself first in certain high-risk groups—men who have sex with men, intravenous drug users, sex workers, mobile populations—and only later moves into the general population. In the early stages of the epidemic, stigma facilitates transmission within high-risk groups, because these already marginalized groups receive little attention from policy makers and the health-care community and are further discriminated against when they are identified with HIV and AIDS [2]. Stigma also prevents or makes it more difficult for members of high-risk groups to access preventive services, including HIV antibody testing [3]. Reidpath and Chan distort this dynamic by describing a model in which stigma leads to fear which leads to unsafe behavior. We know of no one who suggests that stigma causes sex between men or intravenous drug use. Instead, there is evidence that HIV-related stigma makes it difficult for people to take actions to reduce their risks; for example, by accessing HIV education [4], exchanging needles [5], and negotiating condom use [6]. Stigma may even lead women who know they are HIV positive to breast-feed their infants rather than arouse suspicion of their serostatus through formula feeding [7]. This undoubtedly increases the risk of vertical viral transmission.

Reidpath and Chan go on to propose that stigma may actually “slow the spread of infection from those [high-risk] groups to the general population.” Although there is a plausible logic to this suggestion, there is no evidence for it. Even if stigma does reduce the opportunities that marginalized groups have to transmit HIV to the broader population, this would have little effect on the dynamics of a generalized epidemic.

While they recognize that stigma presents a barrier to the treatment and care of people living with HIV, Reidpath and Chan fail to recognize the association this may have with increased transmission. HIV-related stigma discourages people from disclosing their status, entering care, and adhering to antiretroviral regimens, all of which represent missed opportunities for prevention.

Around the world HIV capitalizes on and reinforces social stigma and discrimination, especially the low status of women. Defeating the epidemic requires an honest examination of all these phenomena and interventions that target both the virus itself and its widespread social impacts.
